# Impact of Serotonergic 5HT_1A_ and 5HT_2A_ Receptor Activation on the Respiratory Response to Hypercapnia in a Rat Model of Parkinson’s Disease

**DOI:** 10.3390/ijms25084403

**Published:** 2024-04-17

**Authors:** Kryspin Andrzejewski, Magdalena E. Orłowska, Małgorzata Zaremba, Ilona Joniec-Maciejak, Katarzyna Kaczyńska

**Affiliations:** 1 Department of Respiration Physiology, Mossakowski Medical Research Institute, Polish Academy of Sciences, Pawińskiego 5 St., 02-106 Warsaw, Poland; kandrzejewski@imdik.pan.pl (K.A.); morlowska@imdik.pan.pl (M.E.O.); 2 Department of Experimental and Clinical Pharmacology, Centre for Preclinical Research (CePT), Medical University of Warsaw, 02-091 Warsaw, Poland; mzaremba@wum.edu.pl (M.Z.), ijoniec@wum.edu.pl (I.J.-M.)

**Keywords:** Parkinson’s disease, 6-OHDA model, respiratory disorders, apnea, hypercapnia, serotonin, 5-HT_2A_R, 5-HT_2A_R

## Abstract

In Parkinson’s disease (PD), along with typical motor dysfunction, abnormal breathing is present; the cause of which is not well understood. The study aimed to analyze the effects of stimulation of the serotonergic system with 5-HT_1A_ and 5-HT_2A_ agonists in a model of PD induced by injection of 6-hydroxydopamine (6-OHDA). To model PD, bilateral injection of 6-OHDA into both striata was performed in male Wistar rats. Respiratory disturbances in response to 7% hypercapnia (CO_2_ in O_2_) in the plethysmographic chamber before and after stimulation of the serotonergic system and the incidence of apnea were studied in awake rats 5 weeks after 6-OHDA or vehicle injection. Administration of 6-OHDA reduced the concentration of serotonin (5-HT), dopamine (DA) and norepinephrine (NA) in the striatum and the level of 5-HT in the brainstem of treated rats, which have been associated with decreased basal ventilation, impaired respiratory response to 7% CO_2_ and increased incidence of apnea compared to Sham-operated rats. Intraperitoneal (i.p.) injection of the 5-HT_1A_R agonist 8-OH-DPAT and 5-HT_2A_R agonist NBOH-2C-CN increased breathing during normocapnia and hypercapnia in both groups of rats. However, it restored reactivity to hypercapnia in 6-OHDA group to the level present in Sham rats. Another 5-HT_2A_R agonist TCB-2 was only effective in increasing normocapnic ventilation in 6-OHDA rats. Both the serotonergic agonists 8-OH-DPAT and NBOH-2C-CN had stronger stimulatory effects on respiration in PD rats, compensating for deficits in basal ventilation and hypercapnic respiration. We conclude that serotonergic stimulation may have a positive effect on respiratory impairments that occur in PD.

## 1. Introduction

Parkinson’s disease (PD) is a multisystem disease that primarily targets the dopaminergic nigrostriatal pathway, the progressive neurodegeneration of which yields movement symptoms such as bradykinesia, stiffness, tremor, and postural instability. Another system that can affect the motor and nonmotor aspects of PD and that degenerates in its course is the serotonergic system [1,2]. The ensuing drop in serotonin (5-HT) levels produces abnormal functioning of the serotonergic system, which can result in fatigue, depression, and sleep difficulties, in addition to cognitive, emotional, motor, and breathing dysfunction [1,3]. Serotonin is an important neurotransmitter that modulates the control of breathing and upper airway function [4,5]. 5-HT cells are housed solely in the raphe nuclei of the brainstem, which provide tonic excitatory drive for many components of the respiratory network, such as the pontine and ventral respiratory columns, the Kölliker–Fuse nucleus, the Bötzinger and pre-Bötzinger regions, and the hypoglossal and diaphragmatic motor nuclei [6]. 5-HT is involved in the central chemoreception of CO_2_ as selective destruction of raphe 5-HT nerves results in a reduced response to hypercapnia [7,8,9]. Its genetic deficiency is associated with an increased frequency of central apnea [10]. In addition, serotonin has a well-known stimulating effect on the hypoglossal nerve via activation of 5-HT_2A_ receptors [11].

Among the nonmotor problems experienced by PD patients are breathing disturbances, which include a dysrhythmic breathing pattern, dyspnea, tachypnea, decreased respiratory muscle strength and increased incidence of apnea [12,13,14]. Altered responses to hypoxia and hypercapnia have also been described in both human subjects and animal models of PD [14,15,16].

Currently, to the best of our knowledge, the mechanisms that lead to respiratory deficits or any possible therapeutic approaches that could improve the length and quality of life of patients with PD have not been confirmed. Although L-DOPA, or dopamine agonists, significantly improve the quality of life of PD patients, these drugs can induce adverse effects such as respiratory dyskinesia, tachypnea interspersed with short periods of apnea, pleural fibrosis, dyspnea, pleuritic pain, dry cough, and many others [14,17]. Given that breathing is a complex process, there are great difficulties in understanding the causes of respiratory dysfunction in PD, and there are still unresolved questions to be answered.

Provided that respiratory control is significantly dependent on serotonergic transmission, and the serotonergic system degenerates in Parkinson’s disease, we can hypothesize that the respiratory disorders that occur in PD may be due, at least in part, to changes in the serotonergic system. The rationale for this was shown in our previous study, where damage to the nigrostriatal system by unilateral 6-hydroxydopamine (6-OHDA) administration triggered changes in the serotonergic system, as evidenced by lower 5-HT content in the striatum and brainstem and a reduced ventilatory response to hypoxia following 5-HT_2_ receptor activation [18].

Our goal in the current study was to further explore the role of the serotonergic system in the mediation of the respiratory dysfunction observed in the PD rat model. We used a bilateral model with intrastriatal injection of 6-OHDA, in which a reduced hypercapnic ventilatory response associated with retrotrapezoid nucleus (RTN) degeneration of Phox2b neurons has previously been shown [19]. Our study described the respiratory changes observed in this model during air-breathing and exposure to hypercapnic stimuli and was the first to investigate whether they might be influenced by the pharmacological activation of serotonergic receptors. We targeted 5-HT_1A_ and 5-HT_2A_ receptors because raphe 5-HT_1_ receptor stimulation compromised the hypercapnic ventilatory response [20], and release of 5-HT and postsynaptic 5-HT_2_ receptor activation has been postulated to be critical for central chemosensitivity [21]. We used 8-OH-DPAT, a selective 5-HT_1A_ receptor agonist, and 5HT_2A_ agonists: 25CN-NBOH and TCB-2. To assess the contribution of biogenic amines such as dopamine (DA), noradrenaline (NA) and, most importantly, serotonin (5-HT) to the observed breathing changes, their concentrations in the striatum and brainstem were evaluated.

## 2. Results

### 2.1. Hypercapnic Ventilatory Response (HCVR) in PD and Sham Rats

**Figure 1:**
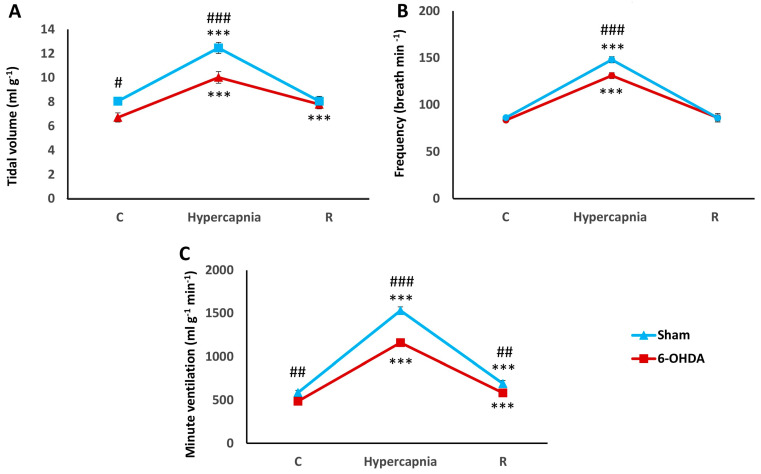
Tidal volume (**A**), frequency of breathing (**B**) and minute ventilation (**C**) during air breathing (normocapnia) and ventilatory response to hypercapnia in Sham (blue line) and 6-OHDA-treated rats (red line). Differences between Sham and 6-OHDA groups were assessed using the Mann-Whitney U test. The Wilcoxon signed-rank test was used to compare the response to hypercapnia and recovery time (R) with control values during normocapnia (C). The data are presented as mean ± SEM; *** *p* < 0.001 vs. normocapnia control value, # *p* < 0.05, ## *p* < 0.01, ### *p* < 0.001 vs. 6-OHDA-treated group (*n* = 20 per group).

Animals in both groups responded to exposure to increased CO_2_ by significantly increasing all respiratory parameters (Figure 1). Reduced baseline tidal volume (V_T_) in 6-OHDA rats resulted in a lower tidal volume ceiling after the hypercapnia stimulus compared to the Sham group (Figure 1A). The opposite was the case with respiratory rate (RR), whose value did not differ between the two groups during breathing ambient air, while its reduced response was observed in 6-OHDA rats after exposure to hypercapnia (Figure 1B). Accordingly, significant reductions in normocapnic and hypercapnic V_T_ (16%) and respiratory rate (20%) during HCVR were reflected in an 18% reduction in baseline minute ventilation (MV) and its significant 36% reduction during HCVR in PD rats compared to the Sham group (Figure 1C).

### 2.2. HCVR in PD and Sham Rats before and after Intraperitoneal (i.p.) Treatment with 8-OH-DPAT

**Figure 2:**
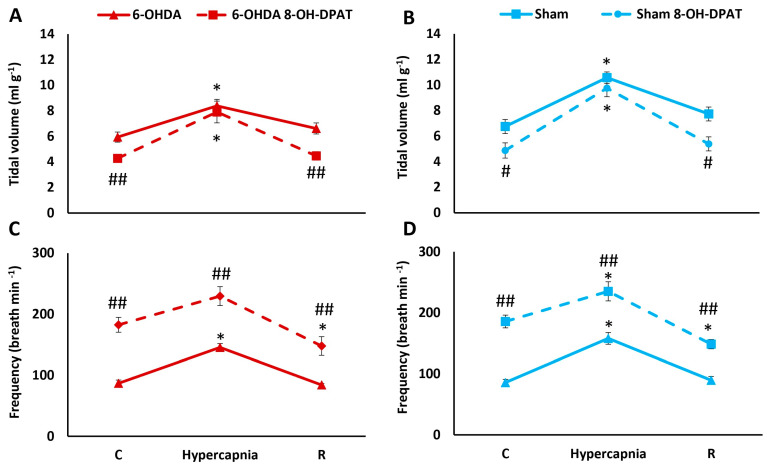
Tidal volume (**A**,**B**) and frequency of breathing (**C**,**D**) during air breathing (normocapnia) and ventilatory response to hypercapnia in Sham (blue line) and 6-OHDA rats (red line) treated with i.p. injection of 8-OH-DPAT (dashed lines). Differences between Sham and 6-OHDA groups were assessed using the Mann-Whitney U test. The Wilcoxon signed-rank test was used to compare the response to hypercapnia and recovery time (R) with control values during normocapnia (C). The data are presented as mean ± SEM; * *p* < 0.05, vs. normocapnia control value, # *p* < 0.05, ## *p* < 0.01, vs. 8-OH-DPAT untreated state (*n* = 6 per group).

Tidal volume after 8-OH-DPAT injection was reduced under normocapnia, while the response to 7% hypercapnia was similar before and after the agonist in both study groups (Figure 2A,B). Administration of 8-OH-DPAT resulted in a more than two-fold increase in RR during ambient air-breathing and respiratory response to hypercapnia in 6-OHDA lesioned and Sham rats (Figure 2C,D).

**Figure 3:**
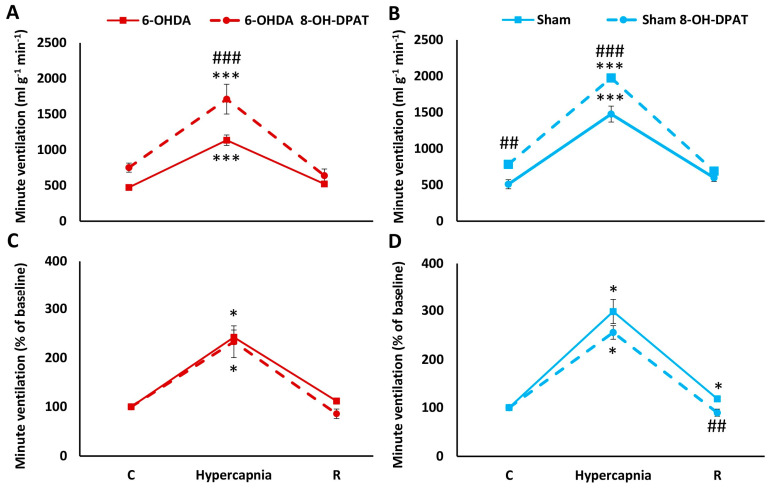
Minute ventilation (**A**,**B**) during air breathing (normocapnia) and ventilatory response to hypercapnia in Sham ((**B**), blue line) and 6-OHDA-treated rats ((**A**), red line) treated with i.p. injection of 8-OH-DPAT (dashed lines). Minute ventilation (**C**,**D**) reactivity to hypercapnia expressed as a percentage of baseline (normocapnia) in Sham ((**D**), blue line) and 6-OHDA-treated rats ((**C**), red line) treated with i.p. injection of 8-OH-DPAT (dashed lines). Two-way ANOVA followed by the Newman-Keuls post hoc test was used to analyze the data presented in panels (**A**,**B**). Data presented in panels (**C**,**D**) were evaluated using the Mann-Whitney U test (differences between Sham and 6-OHDA groups) and the Wilcoxon signed-rank test to compare hypercapnia response and recovery time (R) with control values during normocapnia (C). The data are presented as mean ± SEM; * *p* < 0.05, *** *p* < 0.001 vs. normocapnia control value, ## *p* < 0.01, ### *p* < 0.001, vs. 8-OH-DPAT untreated state (*n* = 6 per group).

Two-way ANOVA revealed the effect of hypercapnia on minute ventilation increases in both 6-OHDA (*F*_(1,2)_ = 61.65, *p* < 0.00001) and Sham rats (*F*_(1,2)_ = 467.59, *p* < 0.00001). 8-OH-DPAT injection had an effect on HCVR response, resulting in increased respiratory response to hypercapnia in PD rats (two-way ANOVA, *F*_(1,2)_ = 13.66, *p* < 0.01), while in Sham rats, 8-OH-DPAT increased both baseline ventilation and HCVR (two-way ANOVA, *F*_(1,2)_ = 21.86, *p* < 0.001) (Figure 3A,B). Additionally, there was a significant interaction effect between hypercapnia exposure and 8-OH-DPAT treatment in PD (two-way ANOVA, *F*_(1,2)_ = 3.64, *p* < 0.05) and Sham rats (two-way ANOVA, *F*_(1,2)_ = 13.80, *p* < 0.001). Nevertheless, the magnitude of the MV response to hypercapnia relative to the control value treated as 100% demonstrated a reduction after 8-OH-DPAT administration only in the Sham group in recovery time after hypercapnia exposure (Figure 3C,D).

### 2.3. HCVR in PD and Sham Rats before and after i.p. Treatment with 25CN-NBOH

**Figure 4:**
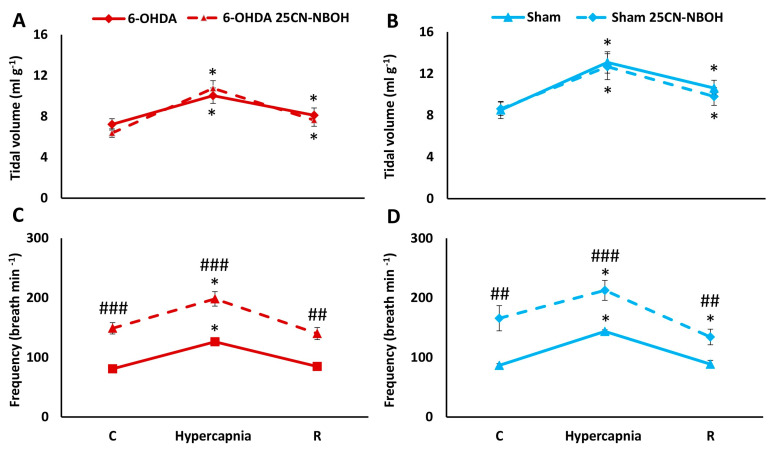
Tidal volume (**A**,**B**) and frequency of breathing (**C**,**D**) during air breathing (normocapnia) and ventilatory response to hypercapnia in Sham (blue line) and 6-OHDA rats (red line) treated with i.p. injection of 25CN-NBOH (dashed lines). Differences between Sham and 6-OHDA groups were assessed using the Mann-Whitney U test. The Wilcoxon signed-rank test was used to compare the response to hypercapnia and recovery time (R) with control values during normocapnia (C). The data are presented as mean ± SEM; * *p* < 0.05, vs. normocapnia control value, ## *p* < 0.01, ### *p* < 0.001, vs. 25CN-NBOH untreated state (*n* = 7 per group).

Exposure to hypercapnia significantly increased the tidal volume and respiratory rate compared to control values in both neurological conditions: 6-OHDA lesioned and Sham (Figure 4). Of the two respiratory pattern parameters, injection of 25CN-NBOH stimulated only the basal normocapnic frequency of breathing, followed by its elevated response to hypercapnia, with no effect on baseline and HCVR tidal volume values (Figure 4A,B). 

**Figure 5:**
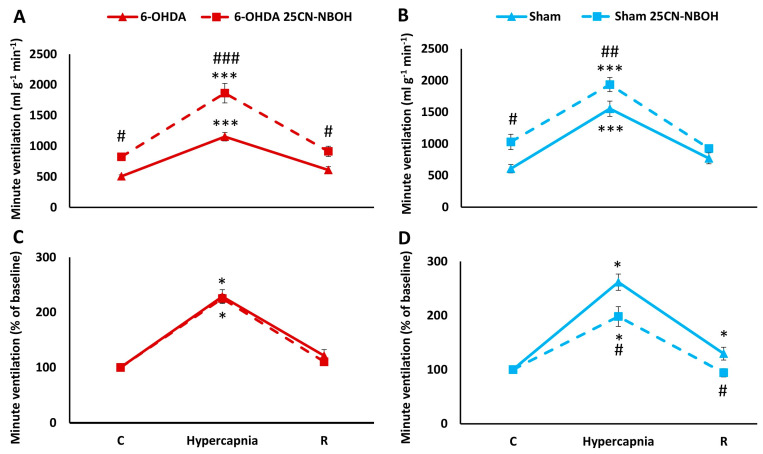
Minute ventilation (**A**,**B**) during air breathing (normocapnia) and ventilatory response to hypercapnia in Sham ((**B**), blue line) and 6-OHDA-treated rats ((**A**), red line) treated with i.p. injection of 8-OH-DPAT (dashed lines). Minute ventilation (**C**,**D**) reactivity to hypercapnia expressed as a percentage of baseline (normocapnia) in Sham ((**D**), blue line) and 6-OHDA-treated rats ((**C**), red line) treated with i.p. injection of 25CN-NBOH (dashed lines). Two-way ANOVA followed by the Newman-Keuls post hoc test was used to analyze the data presented in panels (**A**,**B**). Data presented in panels (**C**,**D**) were evaluated using the Mann-Whitney U test (differences between Sham and 6-OHDA groups) and the Wilcoxon signed-rank test to compare hypercapnia response and recovery time (R) with control values during normocapnia (C). The data are presented as mean ± SEM; * *p* < 0.05, *** *p* < 0.001 vs. normocapnia control value, # *p* < 0.05, ## *p* < 0.01, ### *p* < 0.001, vs. 25CN-NBOH untreated state (*n* = 7 per group).

Two-way ANOVA showed the effect of hypercapnia on minute ventilation increases in both 6-OHDA (*F*_(1,2)_ = 197.94, *p* < 0.00001) and Sham rats (*F*_(1,2)_ = 146.16, *p* < 0.00001). Injection of 25CN-NBOH affected ventilation by increasing baseline minute ventilation and hypercapnia ventilatory response in PD rats (two-way ANOVA, *F*_(1,2)_ = 20.24, *p* < 0.0001) and Sham rats as well (two-way ANOVA, *F*_(1,2)_ = 9.25, *p* < 0.05) (Figure 5A,B). Additionally, there was a significant interaction effect between hypercapnia exposure and 25CN-NBOH treatment only in the PD group (two-way ANOVA, *F*_(1,2)_ = 12.33, *p* < 0.001). MV data expressed as a percentage of change during hypercapnia exposure relative to normocapnic values showed significantly reduced responsiveness to elevated CO_2_ after treatment with 25CN-NBOH only in Sham rats (Figure 5C,D).

### 2.4. HCVR in PD and Sham Rats before and after i.p. Treatment with TCB-2

**Figure 6:**
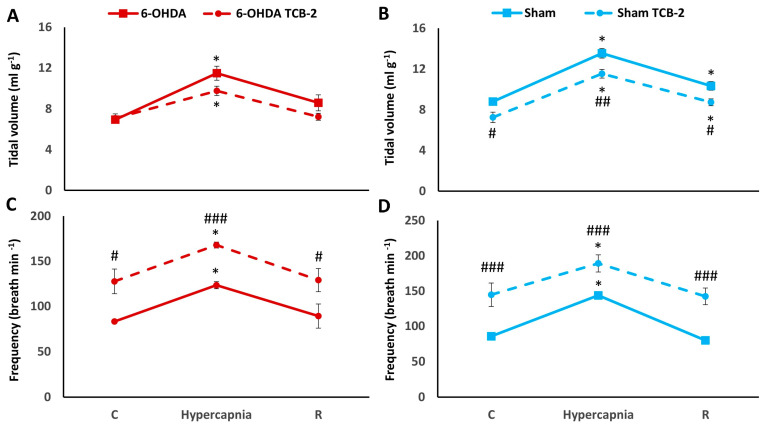
Tidal volume (**A**,**B**) and frequency of breathing (**C**,**D**) during air breathing (normocapnia) and ventilatory response to hypercapnia in Sham (blue line) and 6-OHDA rats (red line) treated with i.p. injection of TCB-2 (dashed lines). Differences between Sham and 6-OHDA groups were assessed using the Mann-Whitney U test. The Wilcoxon signed-rank test was used to compare the response to hypercapnia and recovery time (R) with control values during normocapnia (C). The data are presented as mean ± SEM; * *p* < 0.05, vs. normocapnia control value, # *p* < 0.05, ## *p* < 0.01, ### *p* < 0.001, vs. TCB-2 untreated state (*n* = 7 per group).

After TCB-2, normocapnic tidal volume was significantly reduced only in Sham rats by 18%. While the hypercapnic V_T_ response after TCB-2 was reduced similarly in both groups of rats by 17%, there was a statistically significant difference only in the Sham group (Figure 6A,B). TCB-2 acts mainly by increasing baseline RR with the effect of elevated hypercapnic response of this parameter in 6-OHDA and Sham rats (Figure 6C,D). 

**Figure 7:**
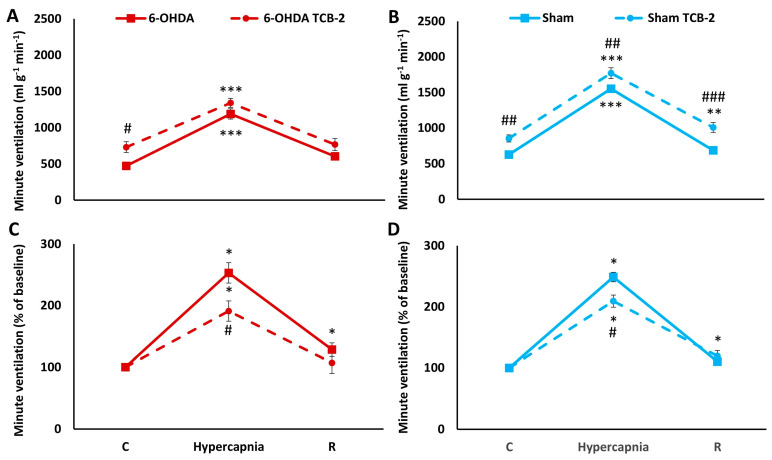
Minute ventilation (**A**,**B**) during air breathing (normocapnia) and ventilatory response to hypercapnia in Sham ((**B**), blue line) and 6-OHDA-treated rats ((**A**), red line) treated with i.p. injection of TCB-2 (dashed lines). Minute ventilation (**C**,**D**) reactivity to hypercapnia expressed as a percentage of baseline (normocapnia) in Sham ((**D**), blue line) and 6-OHDA-treated rats ((**C**), red line) treated with i.p. injection of 25CN-NBOH (dashed lines). Two-way ANOVA followed by the Newman-Keuls post hoc test was used to analyze the data presented in panels (**A**,**B**). Data presented in panels (**C**,**D**) were evaluated using the Mann-Whitney U test (differences between Sham and 6-OHDA groups) and the Wilcoxon signed-rank test to compare hypercapnia response and recovery time (R) with control values during normocapnia (C). The data are presented as mean ± SEM; * *p* < 0.05, ** *p* < 0.01, *** *p* < 0.001, vs. normocapnia control value, # *p* < 0.05, ## *p* < 0.01, ### *p* < 0.001, vs. TCB-2 untreated state (*n* = 7 per group).

Two-way ANOVA showed the effect of hypercapnia on minute ventilation increases in both the 6-OHDA (*F*_(1,2)_ = 112.85, *p* < 0.00001) and Sham group (*F*_(1,2)_ = 358.58, *p* < 0.00001). TCB-2 administration affected minute ventilation, causing a significant increase only in the baseline value in PD rats (two-way ANOVA, *F*_(1,2)_ = 9.40, *p* < 0.01) and in baseline minute ventilation, HCVR response and recovery time in Sham rats (two-way ANOVA, *F*_(1,2)_ = 24.46, *p* < 0.001) (Figure 7A,B), although the course of response to hypercapnia and recovery in PD and Sham rats looks similar. The magnitude of the MV response to hypercapnia relative to the control value taken as 100% indicates that it is reduced after TCB-2 administration in both groups of rats, with a more pronounced effect in 6-OHDA animals, because although the significance level is the same, HCVR decreases in 6-OHDA rats by 25% and in Sham rats by 16%. (Figure 7C,D).

### 2.5. Comparison of HCVR in Sham and PD Rats after i.p. Treatment with 25CN-NBOH, TCB-2, and 8-OH-DPAT

**Figure 8:**
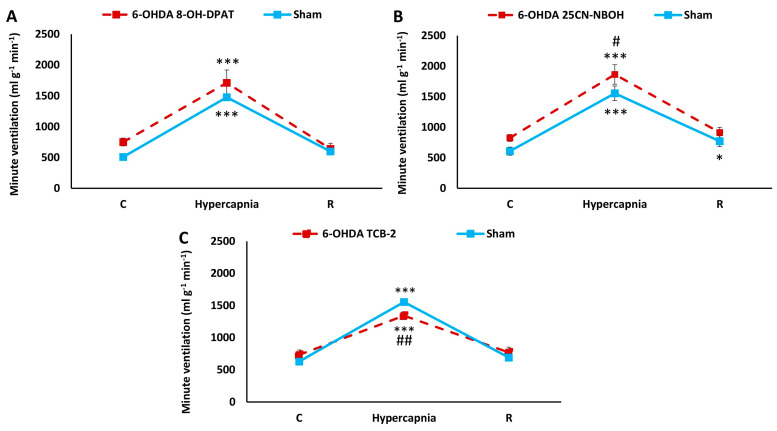
Minute ventilation during air breathing (normocapnia) and ventilatory response to hypercapnia in 6-OHDA treated rats treated with i.p. injection of 8-OH-DPAT (**A**), 25CN-NBOH (**B**), TCB-2 (**C**) (red line, dashed) and Sham untreated rats (blue line). Two-way ANOVA followed by the Newman-Keuls post hoc test was used to analyze the data. C stands for control value; R indicates return after hypercapnia. The data are presented as mean ± SEM; * *p* < 0.05, *** *p* < 0.001, vs. normocapnia control value, # *p* < 0.05, ## *p* < 0.01, vs. Sham untreated rats (*n* = 7 per group).

Two-way ANOVA showed the effect of hypercapnia on minute ventilation increases in the 8-OH-DPAT group (*F*_(1,2)_ = 86.82, *p* < 0.00001), 25CN-NBOH group (*F*_(1,2)_ = 187.05, *p* < 0.00001) and TCB-2 group (*F*_(1,2)_ = 307.40, *p* < 0.00001). Two-way ANOVA also showed a close-to-significant effect of two compounds: 8-OH-DPAT (*F*_(1,2)_ = 3.89, *p* = 0.07) and 25CN-NBOH (*F*_(1,2)_ = 3.66, *p* = 0.07). Additionally, there was a significant interaction effect between hypercapnia exposure and TCB-2 treatment (two-way ANOVA, *F*_(1,2)_ = 12.86, *p* < 0.001). A comparison of basal respiration and MV response to hypercapnia of PD animals after compound injection with that of Sham animals without compounds shows that both 25CN-NBOH and 8-OH-DPAT raise the reduced MV values of 6-OHDA animals to higher levels than observed in control Sham animals (Figure 8A,B). For 25CN-NBOH, the increase in HCVR compared to Sham settles to statistical significance; nevertheless, regardless of the presence of statistical significance, the final effect of both compounds is an elevated minute ventilation above the value present in Sham animals in both normocapnic respiration and during HCVR. In contrast, TCB-2 slightly raised normocapnic MV in PD rats, while it had the opposite effect on HCVR, which was at a significantly lower level, of 16%, compared to the Sham rats (Figure 8C).

### 2.6. Apnea Index Analysis

**Figure 9:**
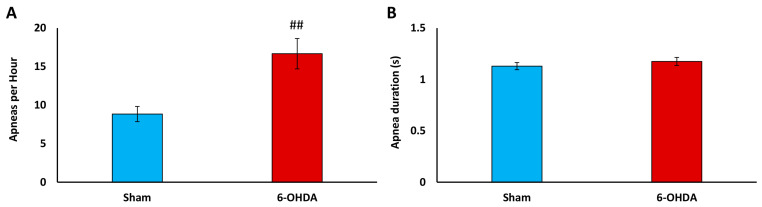
Apnea incidence (**A**) and duration (**B**) in Sham and 6-OHDA-treated rats. Student’s *t*-test was used to analyze differences between 6-OHDA and Sham groups, ## *p* < 0.01, vs. Sham rats (*n* = 8–10 per group).

The apnea analysis showed an increase in the number of apneas calculated per hour in 6-OHDA rats while breathing ambient air (Figure 9). Rats with PD showed 88% more apneas per hour than rats injected with a vehicle. The duration of apneas did not differ between the study groups (1.13 ± 0.03 s vs. 1.18 ± 0.04 s, *p* = 0.63).

### 2.7. Concentration of Biogenic Amines in Striatum and Brainstem

**Figure 10:**
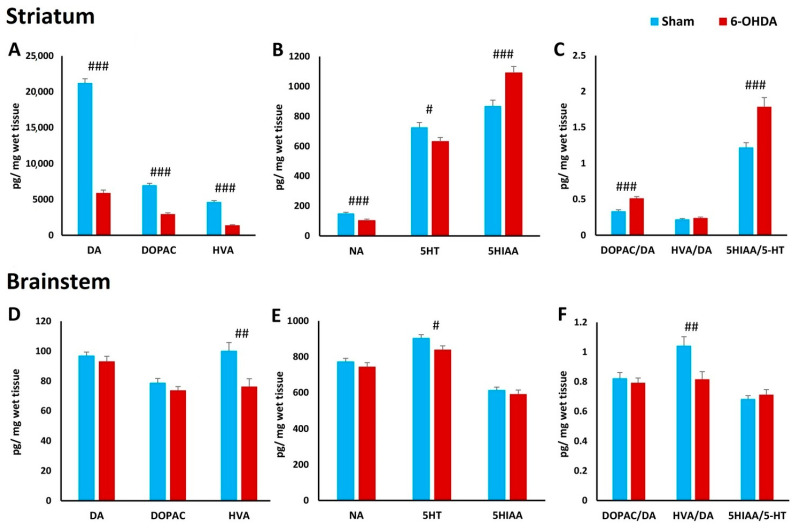
Concentration of biogenic amines in the striatum (**A**–**C**) and brainstem (**D**–**F**) of Sham and 6-OHDA-treated rats. The content of dopamine (DA), noradrenaline (NA), serotonin (5-HT), 3,4-dihydroxyphenylacetic acid (DOPAC), homovanillic acid (HVA) and 5-hydroxyindoleacetic acid (5-HIAA) was assessed by HPLC detection ex vivo and expressed as pg mg^−1^ of fresh tissue. Student’s *t*-test was used to analyze differences between the 6-OHDA and Sham groups. The data are expressed as mean ± SEM. # *p* < 0.05, ## *p* < 0.01, ### *p* < 0.001—significance between both groups (*n* = 16–18 per group).

HPLC analysis confirmed a reduction in monoamine levels in the striatum and brainstem of 6-OHDA-treated rats compared to the Sham group. Administration of neurotoxins induced a 72% decrease in dopamine levels, 32% decrease in noradrenaline and 13% decrease in serotonin content in the striatum (Figure 10A,B). The dopamine metabolites DOPAC and HVA showed lower values in rats treated with 6-OHDA, which was accompanied by an increased turnover of DOPAC/DA (Figure 10A,C). The concentration of serotonin metabolites was increased, which resulted in increased turnover of 5-HIAA/5-HT in the striatum (Figure 10B,C). The only change in the brainstem was an 8% decrease in serotonin and 24% decrease in dopamine metabolite HVA, which corresponded to decreased HVA/DA turnover (Figure 10D–F).

## 3. Discussion

Our study has confirmed that rats in a model of Parkinson’s disease evoked by striatal 6-OHDA injection showed reduced basal ventilation and ventilatory response to hypercapnia, which was previously shown by Tuppy et al. [19]. We also demonstrated that rats with the PD model have more than twice the apnea rate of the Sham group, which, along with reduced HCVR, indicates a central origin of the respiratory disturbance. The reduced basal respiratory drive observed in our 6-OHDA rats can lead to an increased incidence of apnea and a reduced hypercapnic ventilatory response associated with unstable chemosensitivity [22]. The main finding, however, is that both the serotonergic agonists 8-OH-DPAT and 25CN-NBOH had a stimulatory effect on respiration in PD rats, raising its level to that observed in untreated Sham animals and compensating for deficits in HCVR. 

In the central nervous system, serotonin is a neurotransmitter important for many physiological functions, including being involved in the control of breathing, upper airway function, and the pathogenesis of obstructive sleep apnea [5,23]. Ample evidence supports the role of 5-HT neurons that function as central CO_2_ chemosensors [24,25], while not much is known about the involvement of the 5-HT_1A_ and 5-HT_2A_ receptors in the generation or modulation of the hypercapnic ventilatory response. Few data have shown that the application of 5-HT_1A_ or 5-HT_2A_ agonists at the locus coeruleus (LC), which plays some role in HCVR [25], leads to a decline in the ventilatory response to hypercapnia [26]. The application of a 5-HT_1A_ receptor agonist (DOI) and a 5-HT_2A_ antagonist (ketanserin) in an in situ brainstem preparation of juvenile rats abolished the hypercapnic response [21], suggesting an inhibitory effect of the former and an excitatory effect of the latter receptor on the hypercapnia-induced respiratory response. 

Given that serotonin neurons are involved in the control of breathing and the response to hypercapnia, we hypothesized that their loss in PD may negatively affect ventilation and HCVR magnitude, while activation of the 5-HT_1A_ and 5-HT_2A_ receptors may have a beneficial effect. As an example, a selective 5-HT_1A_ receptor agonist was shown to be capable of significantly reducing the number of apneas in a mouse model of Rett syndrome [27]. Recent studies have reported that activation of 5-HT_2A_R facilitated eupnea in mice deficient in serotonin CNS neurons [28] and improved dysregulated breathing after seizures during wakefulness in a mice model of epilepsy [29]. Therefore, we wanted to investigate whether the use of 5-HT_1A_ and 5-HT_2A_ agonists would be effective in reversing the reduced ventilation observed during normocapnic and hypercapnic respiration in 6-OHDA-lesioned animals, which are characterized not only by dopamine but also serotonin deficiency. Analysis of the amine content of the striatum and brainstem showed a significant loss of DA of more than 70% only in the first structure, confirming the effectiveness of inducing the Parkinson’s disease model. This was also accompanied by a statistically significant loss of 5-HT of about 13% in the striatum and 8% in the brainstem.

8-OH-DPAT (8-hydroxy-2-(di-*n*-propylamino) tetralin) is a potent and highly selective 5-HT_1A_ receptor agonist, which has been previously shown to stimulate breathing after intravenous injection in spontaneously breathing anesthetized rats [30] and decerebrated dogs [31]. Subsequently, hyperventilation induced by intravenous administration of 8-OH-DPAT was due to the stimulation of central 5-HT_1A_ receptors of the pre-Bötzinger complex, key respiratory nuclei involved in the generation of respiratory rhythm [32]. Not only that, but even when used systemically, it was able to prevent fentanyl-induced apnea by activating the 5-HT_1A_ receptor located in the caudal–medial region of the nucleus tractus solitarius (NTS) [33]. 

Likewise, the most prominent effect of systemic, specifically intraperitoneal, administration of 8-OH-DPAT observed in our study was tachypnea and hyperventilation, so effective that it raised the reduced ventilation in 6-OHDA rats to a state exceeding that present in the Sham group. The difference between the groups may have been due to greater control ventilation in Sham rats and reaching a certain maximum ceiling effect that prevented a greater increase in ventilation during HCVR, unlike in PD rats. 8-OH-DPAT has been shown to have many beneficial outcomes, such as amelioration of ischemic and traumatic brain injury [34,35] and anti-aggressive, anxiolytic and antidepressant effects [36,37], and together with its central activity after systemic administration [38], it may have a positive impact on the respiratory dysfunction found in PD.

While 5-HT_2A_R agonists are well-known psychedelics, which may limit their potential therapeutic uses, our interest in them stems from the important role of the 5-HT_2A_ receptor in respiratory rhythm generation and normal eupneic respiration [28,29,39,40,41], as well as the modulation of respiratory motor neurons [11,42].

Of the two 5-HT_2A_ receptor agonists tested, only 25CN-NBOH was effective in reversing reduced ventilation in 6-OHDA rats during both normocapnia and respiratory response to hypercapnia. On the other hand, TCB-2 caused a slight increase in basal ventilation in PD rats, but the response to hypercapnia was significantly reduced compared to Sham animals. The discrepancy in the effect of the two compounds may be due to their selectivity toward 5-HT_2A_ receptors. 25CN-NBOH shows high binding affinity to 5-HT_2A_R and strong selectivity for 5-HT_2A_R over 5-HT_2B_R and 5-HT_2C_R in various functional and binding radioligand assays [43], while TCB-2 is not considered as a selective 5-HT_2A_ agonist and its full pharmacological profile has not yet been characterized [44].

The question is whether depletion of dopamine in the striatum and serotonin in the striatum and brainstem can translate into diminished ventilation and respiratory response to hypercapnia.

Significantly greater serotonin losses of 30% in the striatum and 20% in the brainstem were observed in our previous study, which, like the current study, showed reduced basal ventilation. On the other hand, it was a unilateral model with 6-OHDA administration to the medial forebrain bundle, where only the respiratory response to hypoxia was studied [18]. Interestingly, in the latter study, we tested DOI, which significantly increased the basal respiratory rate and minute ventilation in PD animals, corroborating the current results using 25CN-NBOH and TCB-2. However, DOI of all 5-HT_2A_R agonists is not highly selective and has an affinity for 5-HT_2B_R and 5-HT_2C_R [43].

The anatomical changes in the brainstem confirmed earlier in this model may also have contributed to the observed decrease in ventilation and HCVR. Studies in an identical rat model of PD with injection of 6-OHDA into the striatum showed neuronal loss in the most important CO2/H+-sensitive region of the brainstem, such as the retrotrapezoidal nucleus (RTN) and the nucleus tractus solitarius, which is thought to contain chemosensitive areas and to which the RTN projects [19,45]. 

In the same model, neurodegeneration was observed in CO_2_-activated neurons in the periaqueductal gray matter (PAG) that projected indirectly from the substantia nigra pars compacta (SNpc) to the RTN and was correlated with HCVR impairment [46]. It is possible that the reduction in DA-ergic and 5-HT-ergic neurotransmission in the striatum indicative of changes in the SNpc may have affected the projection from the SNpc to chemosensitive areas in our study, as well. The dopaminergic and serotonergic systems interact with each other. Dopaminergic cells in the ventral tegmental area and substantia nigra, and their terminal fields in the paraventricular nucleus, prefrontal cortex and striatum, receive connections from serotonergic raphe nuclei (dorsal and median) [47]. It is also plausible that the reduced concentration of 5-HT in the brainstem present in our study may be due to the degeneration in serotonergic neurons of the raphe nuclei, which are considered to be involved in the hypercapnic ventilatory response (Aung et al., 2024) [23]. Additionally, the RTN, which is crucial in detecting CO2, degenerating in the 6-OHDA model with striatal injection [19], exhibits dense 5-HT innervation, which may provide 5-HT-dependent control of the RTN [4].

Finally, in the striatal 6-OHDA model, astrocyte loss was observed in key regions involved in respiratory activity, such as RTN, NTS and the pre-Bötzinger complex [45]. It is notable that an increase in CO_2_/H + can drive calcium signaling in astrocytes and promote ATP release in regions implicated in respiratory chemosensitivity, while ATP is known to stimulate respiration via activation of P2 purinergic receptors [25,48,49]. Thus, astrocyte loss could potentially also contribute to a reduced HCVR response in this model.

## 4. Material and Methods

### 4.1. Animals

The experiments were approved by the Local Ethics Committee (WAW1/235/2017) and carried out according to the National Institute of Health Guide for the Care and Use of Laboratory Animals and the European Community Council Directive for the Care and Use of Laboratory Animals (86/609/EEC). 

We used young adult male Wistar rats (*n* = 40) weighing 230–260 g (10–12 weeks old) that were bred and housed in the animal vivarium of the Mossakowski Medical Research Institute (Warsaw, Poland). They were held under a 12 h/12 h light/dark cycle and at a controlled temperature of 22 ± 1 °C and 55 ± 10% humidity. The animals also had free access to food and water.

### 4.2. Experimental Groups and Drug Treatment

To induce a model of Parkinson’s disease, twenty rats were injected with 6-hydoxydopamine (6-OHDA) into both striata. The other twenty rats were assigned to a Sham control group, which was injected with 6-OHDA vehicle into the same location in the brain. After treatment, the animals were kept for five weeks under standard laboratory conditions with a 12 h light/12 h dark cycle and unrestricted access to food and water. 

Rats were monitored in a plethysmographic chamber to record control quiet breathing (2–2.5 h) to calculate the number of sleep apneas. The next day, a control hypercapnic test of 7% CO_2_ in O_2_ lasting 3 min was performed, and then the animals were injected intraperitoneally with the following study drugs:

25CN-NBOH at a dose of 1 mg kg^−1^ dissolved in water (Sham *n* = 7, 6-OHDA *n* = 7);
TCB-2 at a dose of 1 mg kg^−1^ dissolved in water (Sham *n* = 7, 6-OHDA *n* = 7);
8-OH-DPAT at a dose of 0.5 mg kg^−1^ dissolved in water (Sham *n* = 6, 6-OHDA *n* = 6).

**Figure 11:**
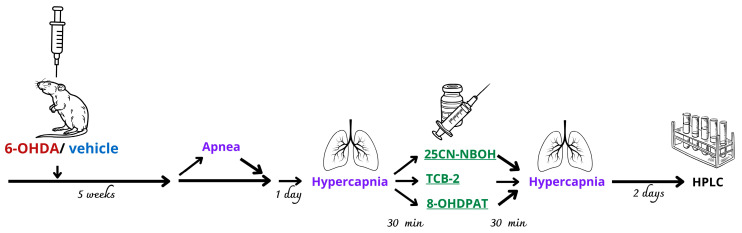
Diagram visualizing animal groups, procedures and treatment on a timeline.

Each 6-OHDA or Sham control group was split into three subgroups, and each subgroup received only one test drug. Thirty minutes after drug injection, the animals were again exposed to the hypercapnic mixture. Two days after the breathing experiments, the rats were anesthetized and the brains (striatum, brainstem) were collected for HPLC analysis (Figure 11).

### 4.3. Surgery and 6-OHDA/Vehicle Injection

Rats were anesthetized with an intramuscular injection of mixture of ketamine (60 mg kg^−1^, Biowet Puławy, Puławy, Poland) and xylazine (6 mg kg^−1^, Biowet Puławy, Puławy, Poland). The anesthetized rats were positioned in a stereotaxic instrument (Digital Lab Standard Stereotaxic Stoelting, Wood Dale, IL, USA). The head of the rat was immobilized to cut the skin and trephine the skull with the use of a dental drill according to stereotaxic coordinates: antero-posterior, bregma: +1.0 mm, lateral: ±2.8 mm, ventral dura: −5.0 mm, and incisor bar: −0.0 mm. Vehicle or 6-OHDA (8 μg per μL dissolved in 0.9% NaCl containing 0.1% ascorbic acid (Sigma Aldrich, Poznań, Poland)) was injected with a sterile Hamilton micro syringe at a volume of 3 μL (rate of 1 μL min^−1^) into the left and right striatum. After the injection, the needle was left in the brain for 5 min to prevent backflow of the solution, and then slowly withdrawn. After the procedure, the rats were given antibiotics and analgesics and then left to convalesce under standard laboratory conditions, with unlimited access to food and water.

### 4.4. Ventilation Measurements

Measurements of ventilation and its responses to hypercapnia (7% CO_2_ in O_2_) and the tested serotonergic agonists were examined in a rodent whole-body plethysmograph (model PLY3223; Buxco Electronix Inc., Wilmington, NC, USA). The pressure difference between the experimental and reference chambers was taken using a differential pressure transducer. The pressure signal was amplified, filtered, recorded and analyzed with data analysis software (Biosystem XA for Windows, SFT3410 230 v2.9; Buxco Electronics, Wilmington, NC, USA) outputting the tidal volume (V_T_, mL) and frequency of breathing (RR, breaths/min). Minute ventilation (MV, mL min^−1^, BTPS) was calculated as the tidal volume multiplied by breathing frequency. V_T_ and MV were normalized to body weight (mL kg^−1^ and mL kg^−1^ min^−1^, respectively). The chamber (4.7 L) was ventilated continuously with atmospheric air at a rate of 2.5 L/min to avoid CO_2_ accumulation. Each rat remained unrestrained in the recording chamber for 30 min for adaptation before measurements of baseline normocapnic parameters. Acute hypercapnia (7%) was achieved by rapid flushing with hypercapnic O_2_-balanced air (5 L). The rectal temperature was taken before and at the end of each experiment. All experiments were carried out at room temperature (22–24 °C). Volume calibration was performed before each experiment by injecting a given volume of air into the chamber.

Parameters of ventilation were recorded 1 min before hypercapnia introduction, during 3 min of hypercapnia and 5 min after the switch to air breathing. The 30 s breathing period preceding hypercapnia and 5 min after hypercapnia was calculated as control normocapnic breathing and return to normocapnic state, respectively. Changes in respiratory parameters from the last two minutes of hypercapnia breathing were averaged. 

### 4.5. Apnea Measurement

The frequency and duration of spontaneous apneas were analyzed in a plethysmographic chamber in which rats were placed for 2–2.5 h during exposure to ambient air. Measurements were taken one day before the experiments with exposure to hypercapnia and serotonergic compounds. Spontaneous apnea was defined as cessation of flow for at least two normal breathing cycles [50]. After the ventilation recording was completed, the number of apneas in a given rat during quiet and regular breathing was calculated. The number of apneas per hour was then calculated.

### 4.6. High-Performance Liquid Chromatography (HPLC) Analysis

Five weeks after the injection of 6-OHDA or vehicle and two days after the end of the hypercapnia and compound administration experiments, the animals were euthanized with an overdose (250 mg kg^−1^) of sodium pentobarbital (Biowet Puławy, Puławy, Poland), and their brains were immediately dissected. The left and right striatum and brainstem were excised. Both striata from the same animal were pooled. Each tissue sample was weighed and frozen (−80 °C) pending further biochemical analysis. The brain tissue was sonicated in a homogenization mixture (containing ice-cold 0.1 M HClO_4_ and 0.05 mM ascorbic acid) and centrifuged (13,000 rpm, 15 min at 4 °C) to separate the proteins. Then, the derived supernatant was filtered (0.2 μm pore size filter; Whatman, Florham Park, NJ, USA). The total tissue contents of dopamine (DA) and its metabolites: 3, 4-dihydroxyphenylacetic acid (DOPAC) and homovanillic acid (HVA), serotonin (5-HT) and its metabolite: 5-hydroxyindoleacetic acid (5-HIAA), and noradrenaline (NA) were analyzed using high-performance liquid chromatography with electro-chemical detection (HPLC-ED) (L-3500 detector; Merck, Darmstadt, Germany) with a glassy carbon working electrode. The electrochemical potential was adjusted to 0.8 V with reference to an Ag/Ag Cl standard electrode. Aliquots (20 μL) were separated on a C—18 column (250 × 4.6 mm reverse phase; Nucleosil, 5 μm, Macherey-Nagel, Düren, Germany). The mobile phase comprised 32 mM sodium phosphate (Sigma-Aldrich, St. Louis, MI, USA), 34 nM citric acid (Sigma-Aldrich, St. Louis, MI, USA) and 1 mM octanesulfonic acid (Sigma-Aldrich, St. Louis, MI, USA), and 54 μM ethylenediaminetetraacetic acid (EDTA; Sigma-Aldrich, St. Louis, MI, USA) was added to 18.3 mΩ of purified water containing 16% methanol (Merck, Darmstadt, Germany). The mobile phase flow rate was maintained at 0.8 mL min^−1^. The samples were quantified by comparison with a standard (Sigma-Aldrich, St. Louis, MI, USA) using Clarity software (version 5.0; DataApex, Prague, Czech Republic) and calibration of an external standard. Monoamine and concomitant metabolite concentrations were further expressed in pg mg^−1^ of fresh tissue.

### 4.7. Statistical Analysis

Statistical analysis was accomplished using STATISTICA 12 software (StatSoft, Krakow, Poland). All data were analyzed using the Shapiro–Wilk test to verify the normality of distribution. Minute ventilation data were analyzed using two-way ANOVA followed by repeated measurements with defined time points (prior to and after hypercapnia) and treatment with serotonergic agents (prior to and after treatment), as a between condition factor, followed by a Newman-Keuls post hoc multiple comparisons test. Because tidal volume and respiratory rate data did not show a normal distribution, they were analyzed using non-parametric tests. Thus, differences between the Sham and 6-OHDA groups were assessed using the Mann-Whitney U test. The Wilcoxon signed-rank test was used to compare hypercapnia response and recovery time with control values in the group. Student’s *t*-test for data with a normal distribution was used to analyze HPLC data and apnea parameters (for two independent 6-OHDA and Sham groups). A confidence limit of *p* < 0.05 was considered statistically significant. The results were expressed as means ± SEM.

## 5. Conclusions

Summarizing, our study showed that depletion of DA and 5-HT in the striatum and 5-HT in the brainstem evoked with striatal 6-OHDA injection translated into reduced basal ventilation, reduced hypercapnic ventilatory response, and increased incidence of apnea. Our studies targeting 5-HT have shown promising results in treating respiratory impairment observed in the PD model. The use of selective serotonergic 5-HT_1A_ and 5-HT_2A_ receptor agonists had a stimulating effect on respiration in PD rats, raising its levels to those observed in untreated Sham animals and compensating for deficits in HCVR.

In the future, it seems of interest to study the effect of influencing the serotonergic system to reduce the number of apneas observed in this model.

## Data Availability

The data used to support the findings of this study are available from the corresponding author upon request.
